# β-Glucan as a Sustainable Alternative to Stabilize Pavement Subgrade

**DOI:** 10.3390/polym14142850

**Published:** 2022-07-13

**Authors:** Vishweshwaran M, Evangelin Ramani Sujatha

**Affiliations:** School of Civil Engineering, SASTRA Deemed University, Thanjavur 613401, India; vishweshwaran36@gmail.com

**Keywords:** biopolymer, geotechnical, pavement subgrade stabilization

## Abstract

Beta glucan (β-Glucan), a polysaccharide biopolymer, is used to improve the subgrade strength of clayey soils in an attempt to advocate a sustainable, carbon-neutral, and eco-friendly stabilizer. A design thickness catalog was developed for a three-layered flexible pavement using 3D finite element analysis (FEA) and layered elastic analysis. The analyses were performed for β-glucan-treated fine-grained soils with varying traffic intensities based on a mechanistic design philosophy conforming to IRC: 37-2018. Genetic programming (GP) was employed to obtain equations governing the rutting and fatigue failure in pavements. Thirty-nine datasets were used in the determination and analysis of critical strains governing the failure of a flexible pavement. Energy-dispersive X-ray spectroscopy (EDS), Fourier transform infrared spectroscopy (FTIR), scanning electron microscopy (SEM), Zetasizer analysis, and pH tests of the β-glucan-treated soil revealed the mechanism of strength improvement of the fine-grained soils. The savings in cost for a 1 km stretch of the pavement were estimated to be 14.3%.

## 1. Introduction

Geomaterials vary both in the spatial and vertical extent to a large degree. Soils with inherently inadequate engineering properties do not always require expensive deep foundation or soil replacement. The governing factors to be considered in the handling of soils with additives include the quantity of carbon dioxide emissions, applicability of the additive for a particular soil, cost of implementation, environmental pollution, etc. Keeping in view sustainability in construction practices, β-glucan, a polysaccharide biopolymer, has been chosen for the improvement of kaolin clay. The present investigation focuses on select index and engineering properties of a thermally treated polysaccharide biopolymer stabilized with fine-grained kaolin clay.

Biopolymers possess the ability to form interconnected networks and gels due to their molecular structure. They have practically replaced synthetic polymers in packaging, industrial, manufacturing, and paper industries, etc. [1]. In the construction sector, β-glucan is used to improve the workability of concrete with a reduction in water content [2]. Pérez et al. (2019) [3] adopted the usage of lignin biopolymer from industrial wastes for improving the surficial layer of pavement, thus offsetting the usage of bitumen. Substitution of bitumen by 20% lignin resulted in improved resistance to moisture and an increased resilient modulus of the bitumen. Lee et al. (2019) [4] improved silty sand using xanthan gum for the construction of pavement shoulder and observed that it improved the unconfined compressive strength (UCS) from 1.1 MPa to 4.9 MPa. Arab et al. (2019) [5] revealed the capability of sodium alginate in pavement subgrade improvement by treating it with fine-grained soils. The addition of sodium alginate in concentrations of 2% for clayey and 4% for silty soils increased the stiffness and brittle behavior of the soils. Utilization of guar gum biopolymer in combination with lime resulted in an improvement in the UCS and California bearing ratio (CBR) of the soil by 650% [6]. Smitha et al. [7] strengthened silty sand with agar biopolymer and improved its ability to withstand liquefaction. Triaxial testing revealed that increase in curing time to 7 days resulted in increase in cohesion by 1700% from 1.6 kPa to 29 kPa. Keshav et al. [8] noted an increase by 630% in the UCS of xanthan gum-amended silty soil after 28 days of curing. Moradi et al. [9] compared the performance of biopolymer stabilization with that of microbial-induced calcium carbonate precipitation (MICP), cationic polyelectrolyte (CPE), and Nicoflok polymer. After 28 days of curing, the results showed the maximum UCS for the β-glucan-treated soil when compared to the other treatment methods. The highly compressible clay soil achieved an UCS of 5.6 MPa on treatment with β-glucan, whereas MICP, CPE, and Nicoflok polymer attained 3.7 MPa, 3.94 MPa, and 4.93 MPa, respectively. The resilient modulus of the β-glucan-treated soil increased to twice that of the control soil sample. Nakamatsu et al. [10] reported the efficacy of carrageenan biopolymer in enhancing the tensile and compressive strength of low compressible clay by 92% and 85%, respectively. Hataf et al. [11] observed that chitosan biopolymer improved the shear strength of soil possessing low plastic clay containing silt and clay. The improvement in cohesion was twice that of the control sample, whereas a negligible increase in the friction angle was observed. Keshav et al. [8] noted a decrease in the free swell index of expansive clayey soil by 45% on treatment with xanthan gum biopolymer. A review of the literature shows that biopolymers have immense potential for subgrade strengthening.

Rutting deformation and fatigue cracking are two of the most severe distresses affecting the structural properties governing a flexible pavement [12]. Rutting of the subgrade is attributed to permanent plastic deformation in the subgrade, whereas fatigue cracking is caused by repeated loading. Mechanistic design entails the determination of limiting strains at individual pavement layers due to repeated tire loading. This design philosophy has been outlined in various international standards, such as Austroads’ AP-T-33, the Indian Road Congress’ (IRC) IRC-37, the National Cooperative Highway Research Program’s (NCHRP) NCHRP 1-37 A, etc. [13,14,15]. Layered elastic analysis and FEA of geomaterials are preferred over traditional CBR-based empirical design due to their rapid, comprehensive distress prediction. Luo and Prozzi [16] studied the variation in critical threshold strains using CIRCLY software and found that increasing the asphalt layer thickness to 250 mm resulted in negligible tensile strains. Arimilli et al. [17] used mechanistic-empirical design to examine traditional and recycled pavement courses using KENPAVE, IITPAVE, and ANSYS and declared that a reduction in strain took place at the bottom of the bituminous layer for cold recycled asphalt layers. Arshad [18] performed mechanistic design on stabilized sub-base layer and improved the its resistance against failure strains. Selvi [19] carried out mechanistic-empirical analysis for lime-treated subgrade soil using M-EPDG software and observed that the rutting deformation on treated subgrade soil was reduced by 200%. Lekha et al. [12] used KENPAVE for damage analysis of arecanut-treated subgrade and demonstrated that the damage due to fatigue life was found to be significantly higher than that due to rutting life.

The need for a sustainable and eco-friendly soil stabilizer for subgrade stabilization is addressed in this study. Owing to their distinct biological and workable attributes, biopolymers are widely used in miscellaneous sectors, such as the pharmaceutical, food, and cosmetics industries, etc. [1,20]. Kaolin, a clay with low subgrade strength, was selected for the study. The utilization of β-glucan biopolymer increased the CBR value under varying curing conditions of the kaolin clay, and, thus, a design thickness catalog was developed for the soaked specimens of the thermally treated kaolin clay. A thickness catalog for β-glucan-stabilized kaolin clay is not available for Indian road conditions conforming to the mechanistic design of IRC: 37-2018. Even though biopolymers have been successfully used in soil strengthening in recent years, their capability in flexible pavement design has not yet been studied in detail. CBR values for 0.5%, 1%, 1.5%, and 2% addition of β-glucan yielded 5.28%, 9.38%, 7.27%, and 4.98%, respectively, after thermally curing the samples for 7 days and soaking the samples for 96 h. GP has served as a promising tool in civil and material engineering, owing to its competence in exploring complex data with minimum time consumption and less uncertainty, and, thus, it has been used to understand the factors governing pavement design [21,22]. From an initial random population comprising model solutions, the model with the highest fitness is determined by successive iterations until convergence is reached. GP models were created using “Eureqa” software, which augments the fitness of distinct population functions at each generation by mutation and crossover processes. They provide insight into the relationship between the dependent critical failure strains and the independent traffic loads, material properties, thickness of the pavement layers, etc. Symbolic regression in GP is used to find the relevant function for the data without assuming the structure of the model.

## 2. Materials and Methods

**Figure 1:**
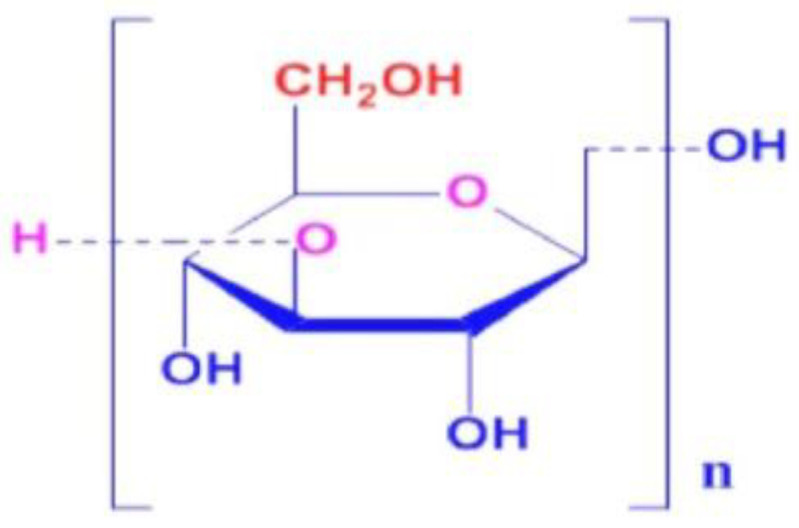
Molecular structure of β-glucan [23].

Commercially available kaolin clay (Astraa Chemicals, Chennai, TN, India) and β-Glucan (Meteroric Life sciences, Ahmedabad, GJ, India) were used for this study. Kaolin is highly plastic in nature, as can be observed from its plasticity index of 34.1%, and falls under the category of highly compressible clay. The molecular weight of β-glucan is 1 million g/mol. β-Glucans are composed of D-glucopyranosyl residues connected through β-(1–3) and β-(1–4) chains. The β-glucan biopolymer possesses elongation strength, adherence, and coating characteristics [1]. The pH values of the clay and β-glucan were observed to be 6.2 and 7.1, respectively. The molecular structure of β-glucan is shown in Figure 1.

Brown-colored yeast β-glucan was used for the study, and it was observed that it did not immediately yield gel formation from 0.5% to 2% concentration [1]. The specimens for CBR testing were placed under three curing conditions. Unsoaked (0 days), thermally cured (7 days), and thermally cured and soaked conditions (11 days) were adopted for the amended clay. The geotechnical properties of the clay are indicated in Table 1.

### 2.1. Sample Preparation

For geotechnical assessment of clayey soils, a wet mixing process was preferred over dry mixing [24]. In wet mixing, β-glucan biopolymer powder and water were added at room temperature and sealed for two hours before mixing with kaolin clay [24,25]. The stabilized kaolin, after mixing with β-glucan, was sealed for three hours before testing. The stabilized kaolin was wrapped tight so that the soil and the β-glucan solution coalesced together by maintaining the soil’s moisture content without any loss. CBR testing was conducted at suitable OMCs for treated and untreated soils under unsoaked, thermally cured, and thermally cured and soaked conditions. The geotechnical tests were conducted by adding β-glucan in concentrations of 0.5%, 1%, 1.5%, and 2% by the dry weight of the kaolin clay.

### 2.2. Methodology

**Figure 2:**
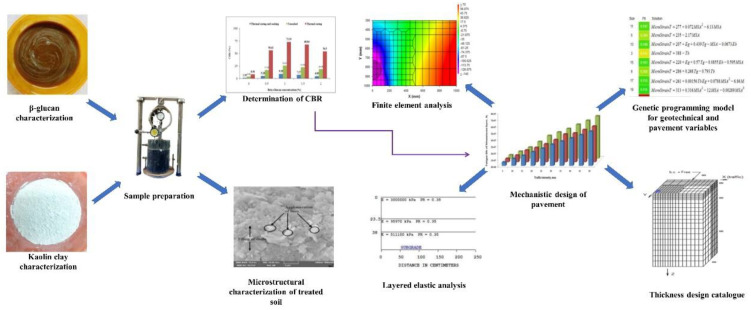
Workflow adopted in this study.

This study utilized thermally cured and soaked CBR in a mechanistic design of the β-glucan-treated pavement. 3D FEA and layered elastic analyses were performed for the same thickness. Cost analysis of the stabilized pavement was included for a 1 km stretch of the pavement. The workflow of this study is presented in Figure 2.

### 2.3. Experimental Program

Determination of the MDU and the corresponding optimum moisture content (OMC) of kaolin and kaolin–β-glucan mixes was carried out in accordance with the procedure outlined in ASTM D698–12e2 [26]. CBR testing was performed as per ASTM D1883–16 [27] to estimate the subgrade strength of the amended and unamended soils. The CBR value is used for the preliminary design of pavements with suitable thicknesses of pavement layers.

### 2.4. Modeling of Flexible Pavement

#### Mechanistic Design

On the basis of design traffic, fatigue cracking on the pavement surface occurs due to horizontal tensile strains (ε_t_) surpassing critical levels at the bottom of the bituminous layer, and rutting is caused by vertical compressive strains (ε_c_) in excess of critical values at the top of the subgrade [14]. Allowable numbers of repetitions for rutting and fatigue were determined based on the strain values of the EverStressFE and KENPAVE softwares. EverStressFE is a 3D FEA software which can analyze pavement structures of up to four layers. KENPAVE was used for three-layered elastic analysis under a circular loaded area. It is capable of designing up to 12 layers and uses the stiffness matrix method for the computation of strains [28]. A wide variety of customizable features are incorporated in KENPAVE which make it versatile for analyses of flexible and rigid pavements. Burmister’s elastic theory was adopted for layered elastic analysis of the treated flexible pavement [29]. Three-layered elastic analysis and FEA were carried out on pavement layers for CBR values of 5%, 7%, and 9% and for traffic intensities of 5, 10, 15, 20, 25, 30, 35, 40, 45, and 50 million standard axles (MSA). Single-axle dual-wheel assembly was selected for the design. The wide array of traffic intensities was selected so as to interpret the effect of traffic loads on pavement design and to determine the interrelationships among them.

The procedure for determining critical strains is as follows:

- Choosing appropriate thicknesses of individual pavement layers;
- Assigning resilient modulus and Poisson’s ratio for all layers;
- Specifying the type of wheel, number of axles, tire pressure, and load per tire;
- Specifying the vertical coordinates where the critical strains are to be evaluated;
- Selection of interface condition as “fully bonded” for all of the layers;
- Trial-and-error approach until a balanced design for rutting and fatigue is obtained.

**Figure 3:**
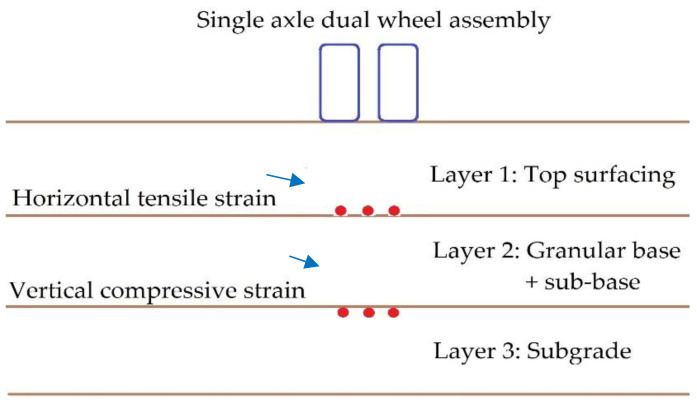
Location of critical strains.

**Figure 4:**
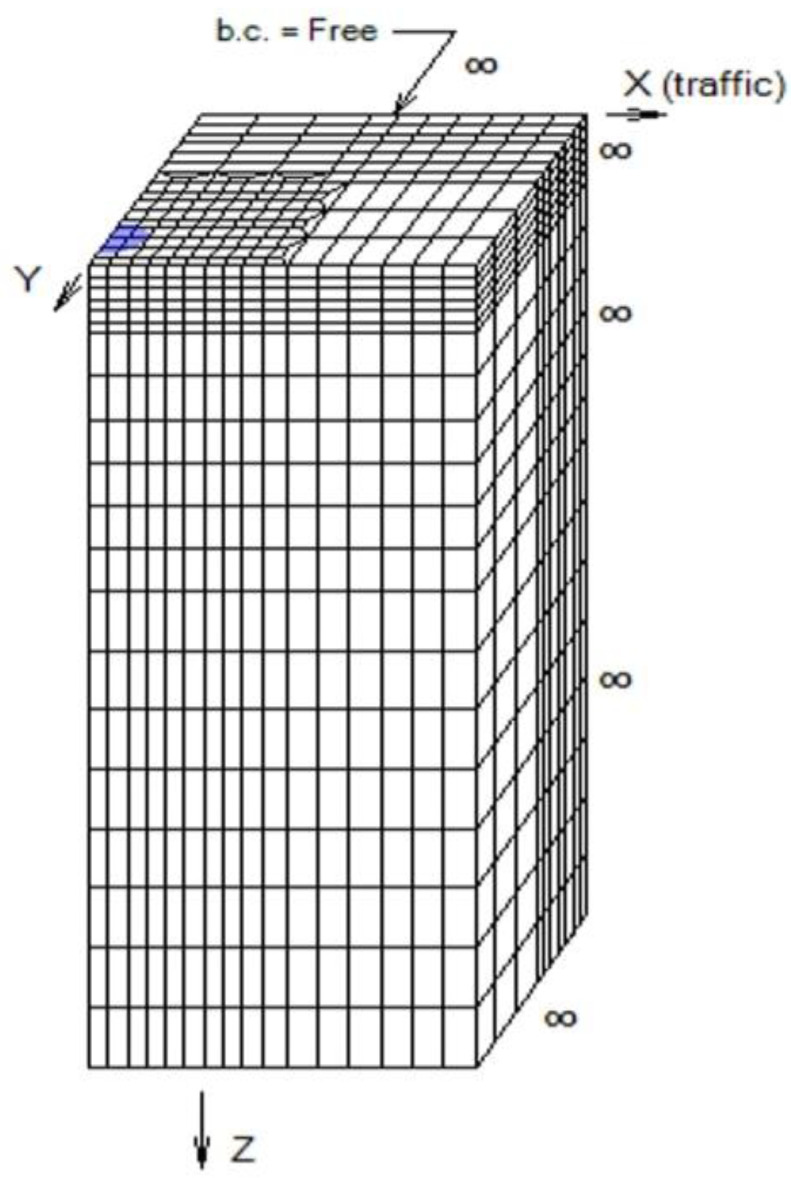
Boundary conditions and meshing of flexible pavement structure.

Figure 3 indicates the cross-section of a flexible pavement and the location of limiting strains at interfaces, and Figure 4 represents the boundary conditions of the FEA model.

Localized meshing was applied around the loaded area with 17,423 nodes and 3800 finite elements. The free boundary condition was chosen for the Y boundary of Layer 1 away from wheel. For both x and y directions, nine elements were chosen for refined region, whereas six elements were selected for the coarse region [30]. The lengths along the x and y directions in the refined region were 400 and 550 mm, respectively. Symmetric meshes with solid quadratic elements comprising 20 nodes were adopted to account for computational time and accuracy [30]. IRC: 37-2018 specifies a minimum subgrade CBR of 5%; the CBR of untreated kaolin clay was found to be 1.39%. The contact pressure, Poisson’s ratio, and resilient modulus of bitumen binder were adopted as specified in IRC: 37-2018. The equivalent modulus of the granular and subgrade layers was adopted based on IRC: 37-2018 [14]. The outputs of the 3D FEA were compressive strains and tensile strains at the subgrade and bituminous surface, respectively.

### 2.5. GP

Rutting and fatigue strain models were analyzed by GP to interpret the factors influencing the failure of the β-glucan-treated flexible pavement. Datasets for the GP models were recorded from the multiple MSA of the mechanistic design. The mathematical models derive equations for rutting and fatigue strains and predict the critical variables governing the structural performance of the pavement. The key feature of symbolic regression is the determination of a mathematical expression inscribed in a symbolic configuration ensuring impeccable fitness of data. To create GP models, a binary tree representing contending functions with terminal and non-terminal nodes was indicated by variables and functions, respectively. The sub-trees undergo the process of mutation and crossover at different generations, leading to a reduction in errors associated with the fitness of the contending functions [31].

### 2.6. Micro-Structural Analyses

EDS, FTIR, SEM, and Zetasizer were the micro-structural tests conducted on kaolin clay and β-glucan-treated kaolin. Macro-variations in engineering properties of the soil arise due to micro-modifications in the structure of the stabilized soil. Tests for pH and viscosity were also conducted to understand the stabilization effects of β-glucan-treated clay.

## 3. Results

### 3.1. Compaction Behavior of β-Glucan-Treated Soil

**Figure 5:**
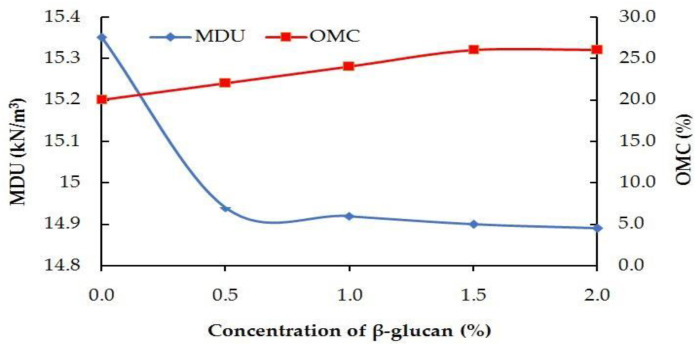
Compaction characteristics of β-glucan-treated soil.

The MDU of the kaolin clay was found to be 15.35 kN/m^3^ for the 20% OMC. A reduction in MDU was observed for the modified kaolin with increasing concentration of β-glucan. The maximum decrease in MDU was observed for 1.5% and 2% concentration of β-glucan. This marginal reduction in MDU from 15.35 kN/m^3^ to 14.89 kN/m^3^ is attributed to the following reasons. The initial bond formation between the clay and the polymer might be disintegrated due to the impact of blows. This could result in a decreased unit weight of the treated soil [32]. The reduction in MDU was noted by Ayeldeen et al. [33], who adopted two polysaccharide biopolymers to stabilize collapsible soil. An increase in the viscosity of the biopolymer gel was found to be the underlying reason for the reduction in MDU. Kaolin could be easily displaced by the β-glucan solution, resulting in reduced MDU. The maximum increase in OMC was obtained for 1.5% and 2% concentration of β-glucan-treated soil. The hydrophilic biopolymer absorbs water from voids of the kaolin and affects the double layer of the clay, and, thus, a greater quantity of water is required by the treated clay [24,34]. As the dosage of β-glucan increased, the quantity of water absorbed by the β-glucan was also greater. Compaction parameters are presented in Figure 5.

### 3.2. Subgrade Strength of β-Glucan-Treated Soil

**Figure 6:**
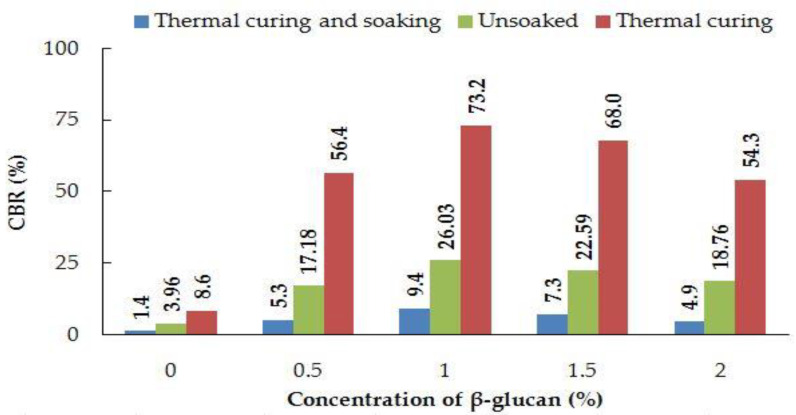
CBR of unsoaked, thermally cured, and thermally cured and soaked samples for untreated and β-glucan-treated kaolin.

Encouraging results in the CBR values of thermally cured specimens underlined the potential of β-glucan in stabilizing the subgrade and prompted the preparation of a design thickness catalog of the treated soil. The CBRs of unsoaked, thermally cured, and thermally cured and soaked kaolin specimens were found to be 3.96%, 8.6%, and 1.39%, respectively. Kaolin clay has exhibited enhanced engineering properties on treatment with xanthan gum biopolymer [35], indicating the possibility of using other polysaccharides such as β-glucan to improve the strength of soil. The CBRs of unsoaked specimens were found to be higher than those of soaked specimens for all β-glucan concentrations, whereas the CBRs of thermally cured specimens exhibited the highest CBR values among all curing conditions. A comparison of CBR results for varying curing conditions is presented in Figure 6.

### 3.3. Micro-Structural Characterization

#### 3.3.1. SEM

**Figure 7:**
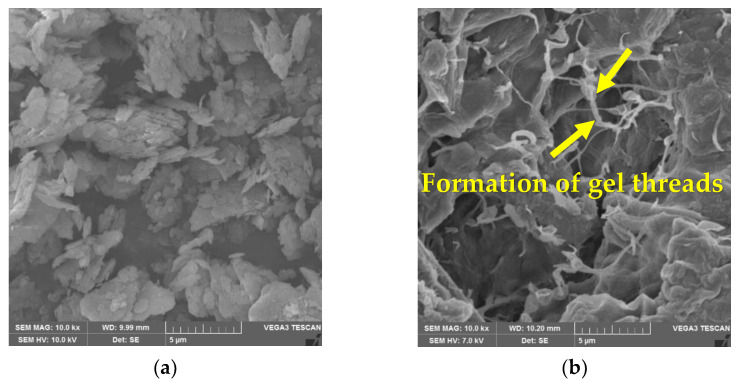
SEM Micrographs. (**a**) Kaolin clay; (**b**) β-glucan-treated kaolin clay.

SEM images were used to observe the topographical changes in the kaolin before and after its treatment with β-glucan. Specimens for micro-structural investigations were extracted from the optimum β-glucan percentage (1%) sample belonging to the CBR test. Figure 7 shows SEM images of failed specimens after the 28th day of sample preparation. β-Glucan-treated kaolin shows the formation of gel threads that stiffen the soil matrix.

#### 3.3.2. FTIR

**Figure 8:**
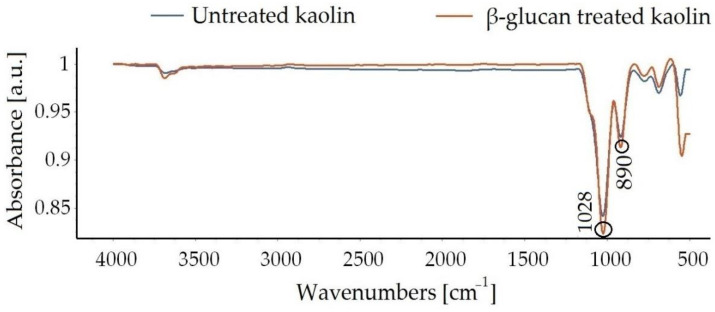
FTIR curves of untreated and β-glucan-treated kaolin clay.

FTIR curves yielded minimal changes for the untreated and the treated soils. Peaks of the amended clay in the absorbance spectrum were observed at 1028 cm^−1^ and 890 cm^−1^, suggesting the presence of β-glucan in the treated kaolin [36]. Figure 8 shows the FTIR curves of kaolin clay and treated kaolin after 7 days of curing.

#### 3.3.3. EDS

**Figure 9:**
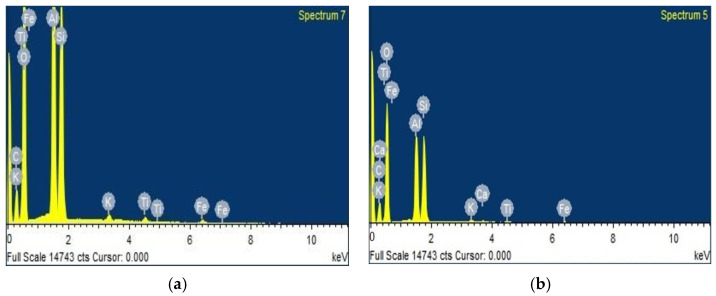
Results of EDS analysis. (**a**) Untreated kaolin; (**b**) β-glucan-treated kaolin.

The EDS spectrum, as observed in Figure 9, indicates that carbon (15.85%), oxygen (60.09%), aluminum (11.31%), and silicon (11.60%) were predominant in kaolin clay, along with traces of iron, titanium, and potassium.

An increase in carbon from 15.85% to 19.27% and a marginal decline in aluminum, oxygen, and silicon were observed for the β-glucan-treated soil. The presence of calcium can aid the formation of the hydration products calcium—alumino—silicate or calcium—alumino—hydrate.

### 3.4. Particle Size Analysis

**Figure 10:**
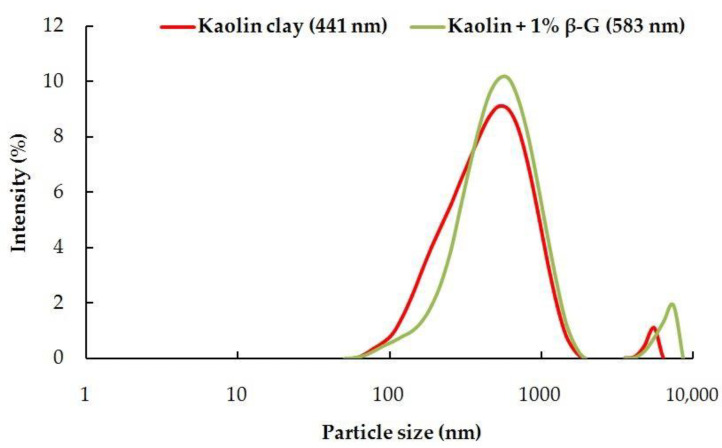
Particle size analysis of untreated and β-glucan-treated kaolin.

A Malvern panalytical Zetasizer was used to analyze the variation in average particle size distribution of untreated and treated kaolin clay. The diameter of the particle size varied from 441 nanometers to 583 nanometers after the 28^th^ day of sample preparation. Figure 10 shows the particle size distribution for the kaolin clay and the β-glucan-modified kaolin clay.

An increase in particle size of the β-glucan-treated clay is an indicator of cementitious aggregation during the first 28 days of curing. This chemical reaction facilitated the blending of kaolin clay and β-glucan, which resulted in the filling of voids and establishment of connecting bridges within the soil as well as external coating on the surface of the treated clay. Application of heat and establishment of chemical bonds improve the adhesion of the kaolin clay particles, which results in increased stiffness and improved CBR.

### 3.5. Mechanistic Empirical Design

#### Comparison of Obtained Strains vs. Allowable Strains

The maximum permissible compressive microstrains (με) and tensile microstrains (με) for 5 MSA traffic intensity were 617.60 με and 266.00 με, respectively. The calculated strains for 0.5%, 1%, 1.5%, and 2% β-glucan-treated soils are within the limiting critical strains as stated in IRC: 37-2018. For any particular traffic condition, KENPAVE underestimates the tensile strains, and EverStressFE underestimates the compressive strains; the design was carried out by considering the severity of the output strains in both software. This variation in the predicted strains was similar to the determination of strains by Arimilli et al. [17], who used the ANSYS and KENPAVE software. Table 2 presents the comparison of target strains and design strains.

### 3.6. Design Thickness Catalog

Thermally cured β-glucan kaolin specimens (1%) resulted in a minimum eligible CBR for the flexible pavement design [14]. It is capable of adsorbing onto the surficial layer of kaolin due to intermolecular interactions [37,38]. It was observed that the increase in binder grade from VG30 to VG40 facilitated the reduction in bituminous layer thickness for the same traffic loading. This is attributed to the increase in the resilient modulus of the bituminous layer from 2000 MPa to 3000 MPa, and, thus, VG40 is recommended for traffic intensity exceeding 20 MSA by IRC: 37-2018 [14]. Table 3 presents the design thickness catalog of the treated soil.

The resilient modulus represents the stiffness of the material when subjected to compressive loading. Numerical investigations revealed that the enhanced stiffness of the treated soil is crucial in reducing the thickness of the subsequent layers above the subgrade soil [17,19]. For a particular traffic intensity, the reduction in elastic modulus of the subgrade directly impacts the thicknesses of its upper layers [19]. The two granular layers below the top bituminous surfacing were regarded as a single layer. The depth of the granular layer was heavily lowered by higher traffic intensities while influencing the resilient modulus of the middle layer.

**Figure 11:**
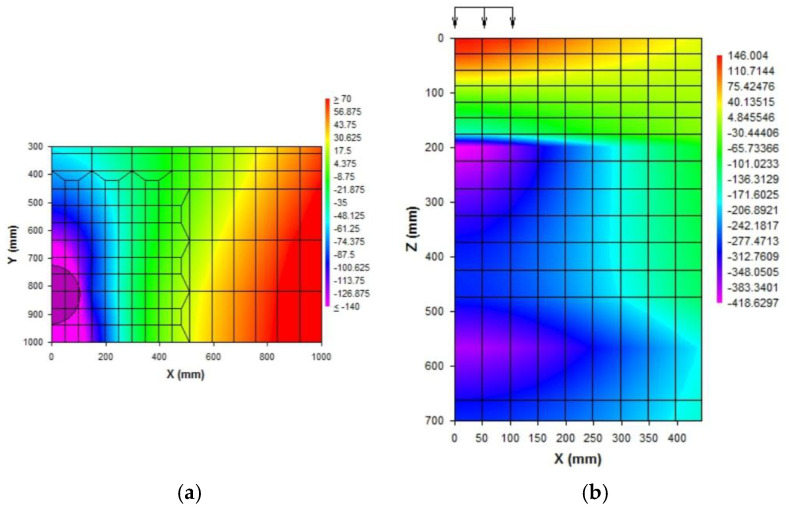
Contour plot of 5% CBR. (**a**) Horizontal strains, 10 MSA; (**b**) vertical strains, 10 MSA.

**Figure 12:**
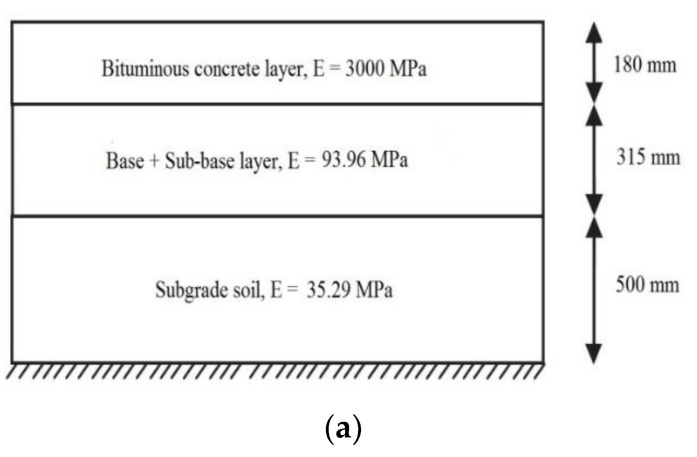

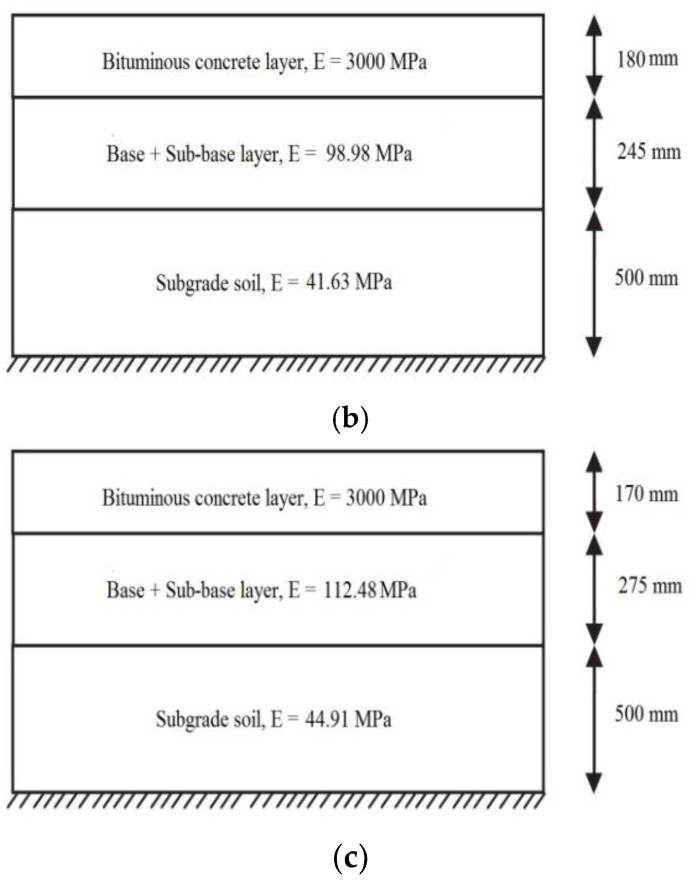
Design thickness. (**a**) CBR = 5%; (**b**) CBR = 7%; (**c**) CBR = 9%.

VG40 and VG30 represent the bitumen binder grades indicating an elastic modulus of 3000 MPa and 2000 MPa, respectively [14]. Control of elastic strains in the subgrade layer allows control of strains throughout the above pavement layers. Owing to the commensurable elastic and plastic strains in pavement layers, the effect of rutting deformation is held in check by the modulus of the middle layer [39]. Figure 11 shows the contour plots of horizontal and vertical strains, respectively, for 10 MSA traffic intensity, and Figure 12 depicts the cross-section of stabilized pavements under three different CBR values for 10 MSA traffic intensity, where it is evident that the thickness of the granular layer decreases with increasing CBR.

### 3.7. GP

The input variables adopted for the GP model were: traffic loading in MSA, CBR, percentage of β-glucan, elastic modulus, and depth of all three layers. The flexible pavement endures inputs in the form of vehicular traffic and produces output in the form of compressive and tensile strains at various depths [18]. There exists a complicated relationship among materials possessing different properties and their ability to withstand moving loads. There is a need to analyze the mechanistic models and the critical factors influencing design life. The influence of the selected factors on the tensile microstrains and compressive microstrains are shown in Equations (1) and (2).

Equation for tensile strain using GP,

(1) $$\varepsilon_{t}=785.555+0.595\mathrm{MSA}-E_{g}-0.064E_{b}-1.556T_{b}$$

Equation for compressive strain using GP,

(2) $$\varepsilon_{c}=1609.798-0.105E_{b}-2.670E_{g}-2.784T_{b}$$

where T_b_, E_b_, and E_g_ indicate thickness of the bituminous layer, elastic modulus of bituminous layer, and modulus of granular layer, respectively.

Equations (1) and (2) indicate that both compressive and tensile strains shall be controlled by choosing the appropriate thickness and elastic modulus of the top two pavement layers. The most important factor controlling pavement design is the traffic intensity in MSA, as observed from both of the equations. Even though there are other variables involved in the design, tensile strain is influenced by the elastic modulus of the top layers, the thickness of the surface layer, and traffic loading, whereas compressive strain is affected only by traffic loading and the thickness of the bituminous surface. The crux of mechanistic philosophy lies in its evaluation of pavement responses for the applied traffic load [39]. The design critical strains decrease with increasing traffic intensity, and, thus, the predicted strains follow the same trend [40,41,42,43]. The statistical performance of the GP models is shown in Table 4.

The data were fragmented automatically into training and validation datasets after random shuffling. Training of the data was performed to obtain efficient models, while assessment of the models on their performance was achieved by validation of the data. Goodness of fit is represented by R^2^, which was found to be 0.99 and 0.98 for rutting and fatigue failures respectively. The mean squared error (MSE) and mean absolute error (MAE) for ε_t_ were found to be 6.195 and 1.284, while the MSE and MAE for ε_c_ were 114.878 and 8.423, respectively.

The predicted critical rutting and fatigue strains were optimized efficiently to ensure adequate serviceability of the pavement design. The model complexity is complementary to the selection of nodes in assigning mathematical functions. To obtain efficient models, increasing complexity yields a greater number of runs and population size. However, the increase in complexity increases computational time; GP does not lead to the generation of black box models [22].

### 3.8. Cost Analysis

The design of a flexible pavement necessitates a minimum CBR of 5% [14]. Kaolin clay does not fulfill this requirement, and, thus, the pavement design is not feasible. The cost of β-glucan biopolymer is INR 250 per kg, and a smaller quantity (1%) of β-glucan increases the CBR value. A traffic loading of 25 MSA was considered for the cost analysis, and the corresponding thicknesses of pavement layers were applied for a stretch of 1 km. Savings in cost were determined to be INR 4,585,034 for a 1 km stretch of the pavement, and the percentage of cost savings was estimated to be 14.3%. Thus, the β-glucan-treated subgrade soil showed a marked reduction in cost and thickness of the pavement. A cost comparative assessment is presented in Table 5, as shown below.

## 4. Discussion

Micrographs in Figure 7 obtained from SEM show that voids in the kaolin were filled by gel threads on addition of β-glucan. The gel threads stiffen the soil matrix, improving the strength of the soil matrix [23]. In addition to the filling of voids, the β-glucan biopolymer adsorbs onto the surface of the kaolin due to molecular affinity and becomes a rigid, monolithic mass with the increasing number of days. The aggregation of clay particles is due to its interaction with the β-glucan molecules, which lead to stable confluence areas [1]. FTIR peaks reveal that the marginal displacements in wavenumber could be associated with the hydrated cementitious kaolin–β-glucan interlocked matrix. The formation of a cemented complex is marked by a strength gain and volume reduction [44,45]. The presence of hydrogen bonds in the clayey matrix limits the swelling potential of kaolin [45]. Figure 9 illustrates the composition of untreated kaolin without the presence of calcium, whereas calcium is detected in the β-glucan-treated clay. Moayedi et al. [46] reported that the presence of calcium ions in treated kaolin clay is responsible for flocculation and aggregation of the soil structure. β-Glucan, being a polysaccharide, is capable of coating the soil, filling the voids in the soil, and agglomerating the soil, which results in a strengthening of the treated kaolin [47,48]. EDS also reveals a borderline decline in the composition of iron and titanium observed for the β-glucan-stabilized kaolin. The control soil and the β-glucan-added soil exhibited specific surface areas of 15.9 m^2^/g and 13.9 m^2^/g, respectively, after the fourth week of stabilization. Particles of kaolin tend to group at vital nodes in the clay–biopolymer complexes, paving the way for the increase in diameter of the particle. The mean diameter of the modified kaolin increased by 32% after 28 days of curing. Anandha Kumar et al. [23] demonstrated the heavy metal attenuation capability of polysaccharide biopolymers on treatment with clays. Hydrogen bond linkages are developed when β-glucan comes into contact with clay, leading to an increase in subgrade strength [49]. Kang et al. [50] found that thermal curing of β-glucan-treated soil at 60 °C increased the compressive strength compared to curing at 20 °C, and the superior performance was attributed to thermosetting induced by heat adsorption. Raising the temperature of the treated soil decreases the residual moisture content and, thus, increases the strength. Based on the CBR findings presented in Figure 6, both the unsoaked and thermally cured CBRs exhibited superior performance when compared to the thermally cured and soaked CBR. The presence of water affects the mechanical properties of the treated kaolin clay even after thermal curing. The flexible pavement design was adopted by considering the soaked condition, which is regarded as the worst performing scenario, as per IRC: 37-2018. The functional groups of a biopolymer are susceptible to variations in pH due to its influence on the charge of the treated soil [34]. The pH of the treated and untreated soils showed negligible variation at all concentrations for all curing days. This facilitated a strength gain with increasing days of curing. Excess biopolymer concentration beyond the optimum dosage tends to push the kaolin particles, which leads to the suppression of electrostatic bonding [50]. Elkafuroy et al. [51] noted that xanthan gum-treated fine sand exhibited a higher CBR in dry conditions when compared to wet conditions. It was also noted that the yeast β-glucan formed gel after a period of 14 days. The gel hardened to a film with an increasing number of days and remained stable for more than 6 months. The soft film was transformed into a rigid barrier after 90 days. β-Glucan biopolymer also formed films on wetting and drying. Anandha Kumar et al. [23] reported a thousand-fold decline in hydraulic conductivity after β-glucan stabilization, which was the result of the coating of β-glucan around the clay and the formation of films in the form of barriers which opposed the entry of water. A decrease in voids and a filling of the voids of the soil establish strong connecting bridges in the β-glucan-treated soil. This leads to an increase in stiffness of the kaolin clay and, thus, reduced deformation, which contributes to a rise in CBR value in all curing conditions. The pH of the treated soil did not vary significantly after the addition of β-glucan up to 2% concentration by weight. The World Health Organization conducted toxicology studies on xanthan gum and guar gum biopolymers and concluded that they did not pose health risks [52]. The micro-structural study indicated that the β-glucan-stabilized clay underwent modifications in the structural, microscopic, and mineralogical characteristics of the kaolin clay.

## 5. Conclusions

The utilization of β-glucan as an additive enhances the CBR of kaolin soil and allows a reduction in the thickness of the bituminous surfacing. It was found that a 1% addition of β-glucan produced the maximum CBR under all curing conditions. A design thickness catalog was prepared for 5%, 7%, and 9% CBR values of the treated soil. The catalog offers structurally reliable values for stabilized soils possessing varying CBRs. The critical strain levels reveal that the mechanistic design has been intensely optimized. The usage of FEA, linear elastic analyses models, and soft computing techniques help in interpreting the complicated behavior of pavement layers with good precision. It was found that traffic intensity was primarily responsible for governing the failure of the pavement. Mechanistic design is better suited to counteract untimely pavement stresses, compared to CBR-based design, by keeping failure strains in check. The flexibility of mechanistic design shall be used for further assessment of roughness measurements of pavements, incorporation of nonlinear analysis, life cycle cost analysis, etc.

## Figures and Tables

**Table 1:** Properties of kaolin clay.

Specific Gravity	Plasticity Index (%)	Soil Classification (ASTM D2487)	OMC * (%)	MDU ^#^ (kN/m^3^)	UCS (kN/m^2^)	CBR (%)
2.65	34.1	CH	20	15.35	140	1.39

* Optimum moisture content, ^#^ maximum dry unit weight.

**Table 2:** Comparison of critical strains.

MSA.	CBR (%)	Amount of β-Glucan Added (%)	Grade of Bitumen	EverStressFE, ε_c_ (με)	EverStressFE, ε_t_ (με)	KENPAVE, ε_c_ (με)	KENPAVE, ε_t_ (με)	Allowable ε_t_ (με) (as per IRC: 37-2018)	Allowable ε_c_ (με) (as per IRC: 37-2018)
5	7	1.5	VG40	573.20	251.00	613.00	238.40	266.00	617.70
10	7	1.5	VG40	483.30	218.00	530.00	206.00	222.60	530.10
15	7	1.5	VG40	422.90	197.00	474.00	184.90	200.50	484.70
20	7	1.5	VG40	390.60	184.00	444.00	172.90	186.20	454.90
25	7	1.5	VG40	358.00	172.00	413.00	161.60	175.90	433.10
30	7	1.5	VG40	341.40	166.00	393.00	155.60	167.80	416.00
35	7	1.5	VG40	325.70	160.00	383.00	150.70	161.30	402.10
40	7	1.5	VG40	311.00	155.00	369.00	145.70	155.90	390.40
45	7	1.5	VG40	300.00	150.00	358.00	141.40	151.20	380.40
50	7	1.5	VG40	300.40	147.00	359.00	138.90	147.20	371.70
5	9	1.0	VG40	554.60	265.10	602.80	264.10	266.00	617.70
10	9	1.0	VG40	473.80	222.50	525.10	217.80	222.60	530.10
15	9	1.0	VG40	414.20	192.40	468.90	187.50	200.50	484.70
20	9	1.0	VG40	399.00	182.80	454.40	178.00	186.20	454.90
25	9	1.0	VG40	376.50	172.50	432.80	168.00	175.90	433.10
30	9	1.0	VG40	358.80	163.30	415.80	159.30	167.80	416.00
35	9	1.0	VG40	342.40	154.10	400.00	154.00	161.30	402.10
40	9	1.0	VG40	330.00	152.70	388.00	149.40	155.90	390.40
45	9	1.0	VG40	321.00	148.40	379.20	145.40	151.20	380.40
50	9	1.0	VG40	312.20	144.50	370.70	141.50	147.20	371.70
5	5	0.5	VG40	543.10	265.80	610.60	262.00	266.00	617.70
10	5	0.5	VG40	452.70	220.30	525.80	214.80	222.60	530.10
15	5	0.5	VG40	404.90	195.30	480.90	190.40	200.50	484.70
20	5	0.5	VG40	376.00	175.80	453.70	134.60	186.20	454.90
25	5	0.5	VG40	352.30	162.40	430.60	159.00	175.90	433.10
30	5	0.5	VG40	336.50	156.50	414.70	153.80	167.80	416.00
35	5	0.5	VG40	321.50	151.50	399.70	148.80	161.30	402.10
40	5	0.5	VG40	309.90	146.40	388.10	144.50	155.90	390.40
45	5	0.5	VG40	301.20	142.30	379.30	134.50	151.20	380.40
50	5	0.5	VG40	292.90	138.30	370.80	137.00	147.20	371.70
5	7	1.5	VG30	562.50	263.00	602.00	247.90	266.00	617.70
10	7	1.5	VG30	484.40	220.00	529.00	206.70	222.60	530.10
15	7	1.5	VG30	433.30	197.00	481.00	185.50	200.50	484.70
5	9	1.0	VG30	568.20	260.60	614.90	254.60	266.00	617.70
10	9	1.0	VG30	475.40	216.20	525.80	210.60	222.60	530.10
15	9	1.0	VG30	430.10	191.90	482.30	187.30	200.50	484.70
5	5	0.5	VG30	552.00	259.00	617.40	252.20	266.00	617.70
10	5	0.5	VG30	458.80	213.40	528.30	208.40	222.60	530.10
15	5	0.5	VG30	417.50	189.10	484.60	185.10	200.50	484.70

**Table 3:** Design thickness catalogue of β-glucan-stabilized pavement.

Traffic Intensity, MSA	CBR (%)	Amount of β-Glucan Added (%)	Modulus of Base + Sub-Base (MPa)	Modulus of Subgrade (MPa)	Thickness of Surface Course (mm)	Thickness of Base + Sub-Base Course (mm)	Grade of Bitumen
5	7	1.5	98.98	41.63	160	245	VG40
10	7	1.5	98.98	41.63	180	245	VG40
15	7	1.5	99.89	41.63	195	250	VG40
20	7	1.5	99.89	41.63	205	250	VG40
25	7	1.5	100.78	41.63	215	255	VG40
30	7	1.5	101.67	41.63	220	260	VG40
35	7	1.5	102.54	41.63	225	265	VG40
40	7	1.5	103.41	41.63	230	270	VG40
45	7	1.5	103.41	41.63	235	270	VG40
50	7	1.5	99.89	41.63	240	250	VG40
5	9	1	116.97	44.91	145	300	VG40
10	9	1	112.48	44.91	170	275	VG40
15	9	1	106.78	44.91	195	245	VG40
20	9	1	102.77	44.91	205	225	VG40
25	9	1	100.69	44.91	215	215	VG40
30	9	1	97.46	44.91	225	200	VG40
35	9	1	98.55	44.91	230	205	VG40
40	9	1	98.55	44.91	235	205	VG40
45	9	1	97.46	44.91	240	200	VG40
50	9	1	96.36	44.91	245	195	VG40
5	5	0.5	97.88	35.29	150	345	VG40
10	5	0.5	93.96	35.29	180	315	VG40
15	5	0.5	90.52	35.29	200	290	VG40
20	5	0.5	83.91	35.29	220	245	VG40
25	5	0.5	79.12	35.29	235	215	VG40
30	5	0.5	79.94	35.29	240	220	VG40
35	5	0.5	80.75	35.29	245	225	VG40
40	5	0.5	80.75	35.29	250	225	VG40
45	5	0.5	79.94	35.29	255	220	VG40
50	5	0.5	79.12	35.29	260	215	VG40
5	7	1.5	103.51	41.63	195	215	VG30
10	7	1.5	93.33	41.63	230	165	VG30
15	7	1.5	82.85	41.63	250	150	VG30
5	9	1	79.37	44.91	195	195	VG30
10	9	1	88.15	44.91	230	160	VG30
15	9	1	77.45	44.91	255	120	VG30
5	5	0.5	81.56	35.29	205	230	VG30
10	5	0.5	75.72	35.29	240	195	VG30
15	5	0.5	67.29	35.29	265	150	VG30

**Table 4:** Statistical parameters for ε_t_ and ε_c_.

Statistical Parameters	Horizontal Tensile Strain, ε_t_ (με)	Vertical Compressive Strain, ε_c_ (με)
Coefficient of Determination, R^2^	0.99	0.98
Maximum Error	12.410	26.153
Mean Squared Error (MSE)	6.195	114.878
Mean Absolute Error (MAE)	1.284	8.423
Coefficients	4	4
Complexity	15	13

**Table 5:** Cost analysis of 5% and 9% CBR pavements.

S.No.	CBR of Soil	Cost of Bituminous Surfacing (INR)	Cost of Granular Layer (INR)	Transportation Cost of Granular Layer (INR50/m^3^)	Transportation Cost of Subgrade Soil (INR)	Total Cost (INR)
1	1.39%	29,567,732	2,156,361	102,188	137,500	31,963,781
2	9%	26,487,760	850,674	40,313	-	27,378,747

## Data Availability

The data presented in this study are available on request from the corresponding author.

## References

[1] Kasapis S., Norton I.T., Johan B. (2009). Modern Biopolymer Science: Bridging the Divide between Fundamental Treatise and Industrial Application.

[2] Kiho T., Sakushima M., Wang S., Nagai K., Ukai S. (1991). Polysaccharides in Fungi. XXVI. Two Branched BETA -D-Glucans from Hot Water Extract of Yu Er. Chem. Pharm. Bull..

[3] Pérez I.P., Rodríguez Pasandín A.M., Pais J.C., Alves Pereira P.A. (2019). Use of Lignin Biopolymer from Industrial Waste as Bitumen Extender for Asphalt Mixtures. J. Clean. Prod..

[4] Lee S., Chung M., Park H.M., Song K.-I., Chang I. (2019). Xanthan Gum Biopolymer as Soil-Stabilization Binder for Road Construction Using Local Soil in Sri Lanka. J. Mater. Civ. Eng..

[5] Arab M.G., Mousa R.A., Gabr A.R., Azam A.M., El-Badawy S.M., Hassan A.F. (2019). Resilient Behavior of Sodium Alginate–Treated Cohesive Soils for Pavement Applications. J. Mater. Civ. Eng..

[6] Onah H.N., Nwonu D.C., Ikeagwuani C.C. (2022). Feasibility of Lime and Biopolymer Treatment for Soft Clay Improvement: A Comparative and Complementary Approach. Arab. J. Geosci..

[7] Smitha S., Rangaswamy K., Keerthi D.S. (2021). Triaxial Test Behaviour of Silty Sands Treated with Agar Biopolymer. Int. J. Geotech. Eng..

[8] Keshav N., Prabhu A., Kattimani A., Dharwad A., Kallatti C., Mahalank S. (2021). Enhancing the Properties of Expansive Soil Using Biopolymers—Xanthan Gum and Guar Gum. Proceedings of the Indian Geotechnical Conference 2019.

[9] Moradi G., Shafaghatian S., Katebi H. (2022). Effect of Chemical and Biological Stabilization on the Resilient Modulus of Clay Subgrade Soil. Int. J. Pavement Res. Technol..

[10] Nakamatsu J., Kim S., Ayarza J., Ramírez E., Elgegren M., Aguilar R. (2017). Eco-Friendly Modification of Earthen Construction with Carrageenan: Water Durability and Mechanical Assessment. Constr. Build. Mater..

[11] Hataf N., Ghadir P., Ranjbar N. (2018). Investigation of Soil Stabilization Using Chitosan Biopolymer. J. Clean. Prod..

[12] Lekha B.M., Goutham S., Shankar A.U.R. (2015). Evaluation of Lateritic Soil Stabilized with Arecanut Coir for Low Volume Pavements. Transp. Geotech..

[13] Jameson G. (2013). Technical Basis of Austroads Guide to Pavement Technology Part 2: Pavement Structural Design.

[14] Indian Roads Congress (2018). IRC 37: Guidelines for the Design of Flexible Pavements.

[15] Mulandi J., Khanum T., Hossain M., Schieber G. (2006). Comparison of pavement design using AASHTO 1993 and NCHRP mechanistic-empirical pavement design guides. Airfield and Highway Pavement: Meeting Today’s Challenges with Emerging Technologies.

[16] Luo R., Prozzi J.A. (2007). Effect of Measured Three-Dimensional Tire–Pavement Contact Stress on Pavement Response at Asphalt Surface. Transp. Res. Rec..

[17] Arimilli S., Nagabhushana M.N., Jain P.K. (2018). Comparative Mechanistic-Empirical Analysis for Design of Alternative Cold Recycled Asphalt Technologies with Conventional Pavement. Road Mater. Pavement Des..

[18] Arshad M. (2019). Development of a Correlation between the Resilient Modulus and CBR Value for Granular Blends Containing Natural Aggregates and RAP/RCA Materials. Adv. Mater. Sci. Eng..

[19] Selvi P. (2015). Fatigue and Rutting Strain Analysis on Lime Stabilized Subgrades to Develop a Pavement Design Chart. Transp. Geotech..

[20] Karim A.A., Bhat R. (2009). Fish Gelatin: Properties, Challenges, and Prospects as an Alternative to Mammalian Gelatins. Food Hydrocoll..

[21] Sreekanth J., Datta B. (2011). Comparative Evaluation of Genetic Programming and Neural Network as Potential Surrogate Models for Coastal Aquifer Management. Water Resour Manag..

[22] Gandomi A.H., Alavi A.H., Ryan C. (2015). Handbook of Genetic Programming Applications.

[23] Anandha Kumar S., Sujatha E.R. (2021). An Appraisal of the Hydro-Mechanical Behaviour of Polysaccharides, Xanthan Gum, Guar Gum and β–glucan Amended Soil. Carbohydr. Polym..

[24] Rezaeimalek S., Nasouri R., Huang J., Bin-Shafique S. (2018). Curing Method and Mix Design Evaluation of a Styrene-Acrylic Based Liquid Polymer for Sand and Clay Stabilization. J. Mater. Civ. Eng..

[25] Chang I., Im J., Prasidhi A.K., Cho G.-C. (2015). Effects of Xanthan Gum Biopolymer on Soil Strengthening. Constr. Build. Mater..

[26] (2012). Standard Test Methods for Laboratory Compaction Characteristics of Soil using Standard Effort.

[27] (2016). Standard Test Method for California Bearing Ratio (CBR) of Laboratory-Compacted Soils.

[28] Lekha B.M., Ravi Shankar A.U., Sarang G. (2013). Fatigue and Engineering Properties of Chemically Stabilized Soil for Pavements. Indian Geotech. J..

[29] Burmister D.M. (1945). The General Theory of Stresses and Displacements in Layered Systems. I. J. Appl. Phys..

[30] Jiang X., Zeng C., Gao X., Liu Z., Qiu Y. (2019). 3D FEM Analysis of Flexible Base Asphalt Pavement Structure under Non-Uniform Tyre Contact Pressure. Int. J. Pavement Eng..

[31] Koza J.R. (1994). Genetic Programming II.

[32] Ng C.W.W., So P.S., Lau S.Y., Zhou C., Coo J.L., Ni J.J. (2020). Influence of Biopolymer on Gas Permeability in Compacted Clay at Different Densities and Water Contents. Eng. Geol..

[33] Ayeldeen M.K., Negm A.M., El Sawwaf M.A. (2016). Evaluating the Physical Characteristics of Biopolymer/Soil Mixtures. Arab. J. Geosci..

[34] Kwon Y.M., Chang I., Lee M., Cho G. (2019). Geotechnical Engineering Behavior of Biopolymer-Treated Soft Marine Soil. Geomech. Eng..

[35] Saha D., Bhattacharya S. (2010). Hydrocolloids as Thickening and Gelling Agents in Food: A Critical Review. J. Food Sci. Technol..

[36] Galichet A., Sockalingum G.D., Belarbi A., Manfait M. (2001). FTIR spectroscopic analysis of Saccharomyces cerevisiae cell walls: Study of an anomalous strain exhibiting a pink-colored cell phenotype. FEMS Microbiol. Lett..

[37] Theng B.K.G. (1982). Clay-Polymer Interactions: Summary and Perspectives. Clays Clay Miner..

[38] Rao Y. (2007). Gelatin–Clay Nanocomposites of Improved Properties. Polymer.

[39] Huang Y.H. (1993). Pavement Analysis and Design.

[40] Asphalt Institute (1970). Asphalt Institute Thickness Design Manual (MS-1).

[41] Otte E., Savage P.F., Monismith C.L. (1982). Structural Design of Cemented Pavement Layers. Transp. Engrg. J..

[42] Siddique A., Rajbongshi B. An analytical study on design and analysis of stabilised rural roads. Proceedings of the Eastern Asia Society for Transportation Studies.

[43] Ekwulo E.O., Eme D.B. (2009). Fatigue and rutting strain analysis of flexible pavements designed using CBR methods. Afr. J. Environ. Sci. Technol..

[44] Lav A.H., Lav M.A. (2000). Microstructural Development of Stabilized Fly Ash as Pavement Base Material. J. Mater. Civ. Eng..

[45] Al-Swaidani A., Hammoud I., Meziab A. (2016). Effect of Adding Natural Pozzolana on Geotechnical Properties of Lime-Stabilized Clayey Soil. J. Rock Mech. Geotech. Eng..

[46] Moayedi H., Mosallanezhad M. (2017). Physico-Chemical and Shrinkage Properties of Highly Organic Soil Treated with Non-Traditional Additives. Geotech. Geol. Eng..

[47] Kumari N., Mohan C., Nascimento G.M.D. (2021). Basics of Clay Minerals and Their Characteristic Properties. Basics of Clay Minerals.

[48] Azzaroni O., Szleifer I. (2017). Polymer and Biopolymer Brushes: For Materials Science and Biotechnology 2 Volume Set.

[49] Chang I., Prasidhi A.K., Joo G.W., Cho G.C. An Alternative Method for Soil Treatment Using Environmentally—Friendly Biopolymer. Proceedings of the Advances in Civil, Environmental, and Materials Research (ACEM’ 12), World Congress.

[50] Kang X., Bate B., Chen R.-P., Yang W., Wang F. (2019). Physicochemical and Mechanical Properties of Polymer-Amended Kaolinite and Fly Ash–Kaolinite Mixtures. J. Mater. Civ. Eng..

[51] Elkafoury A., Azzam W. (2021). Utilize Xanthan Gum for Enhancing CBR Value of Used Cooking Oil-Contaminated Fine Sand Subgrade Soil for Pavement Structures. Innov. Infrastruct. Solut..

[52] Biju M.S., Arnepalli D.N. (2020). Effect of Biopolymers on Permeability of Sand-Bentonite Mixtures. J. Rock Mech. Geotech. Eng..

